# Full Genome Characterization of Human Influenza A/H3N2 Isolates from Asian Countries Reveals a Rare Amantadine Resistance-Conferring Mutation and Novel PB1-F2 Polymorphisms

**DOI:** 10.3389/fmicb.2016.00262

**Published:** 2016-03-07

**Authors:** Hassan Zaraket, Hiroki Kondo, Akinobu Hibino, Ren Yagami, Takashi Odagiri, Nobuhiro Takemae, Ryota Tsunekuni, Takehiko Saito, Japanese Influenza Collaborative Study Group, Yi Yi Myint, Yadanar Kyaw, Khin Yi Oo, Htay Htay Tin, Nay Lin, Nguyen Phuong Anh, Nguyen Le Khanh Hang, Le Quynh Mai, Mohd R. Hassan, Yugo Shobugawa, Julian Tang, Ghassan Dbaibo, Reiko Saito

**Affiliations:** ^1^Department of Pathology, Immunology, and Microbiology, Faculty of Medicine American University of BeirutBeirut, Lebanon; ^2^Center for Infectious Disease Research, Faculty of Medicine American University of BeirutBeirut, Lebanon; ^3^Division of International Health (Public Health), Graduate School of Medical and Dental Sciences, Niigata UniversityNiigata, Japan; ^4^Influenza and Prion Disease Research Center, National Institute of Animal Health, National Agriculture and Food Research OrganizationIbaraki, Japan; ^5^Department of Traditional MedicineNay Pyi Taw, Myanmar; ^6^Sanpya HospitalYangon, Myanmar; ^7^National Health LaboratoryYangon, Myanmar; ^8^Pyinmana Township HospitalNay Pyi Taw, Myanmar; ^9^National Institute of Hygiene and EpidemiologyHanoi, Vietnam; ^10^Department of Community Health, Faculty of Medicine, UKM Medical CentreKuala Lumpur, Malaysia; ^11^Clinical Microbiology, University Hospitals LeicesterLeicester, UK; ^12^Department of Infection, Immunity and Inflammation, University of LeicesterLeceister, UK; ^13^Division of Pediatric Infectious Diseases, Department of Pediatrics and Adolescent Medicine and the Center for Infectious Diseases Research, American University of Beirut Medical CenterBeirut, Lebanon

**Keywords:** influenza A/H3N2, full-genome, phylogenetic analysis, antiviral, vaccine, evolution, reassortment, PB1-F2

## Abstract

Influenza A viruses evolve at a high rate requiring continuous monitoring to maintain the efficacy of vaccines and antiviral drugs. We performed next generation sequencing analysis of 100 influenza A/H3N2 isolates collected in four Asian countries (Japan, Lebanon, Myanmar, and Vietnam) during 2012–2015. Phylogenetic analysis revealed several reassortment events leading to the circulation of multiple clades within the same season. This was particularly evident during the 2013 and 2013/2014 seasons. Importantly, our data showed that certain lineages appeared to be fitter and were able to persist into the following season. The majority of A/H3N2 viruses continued to harbor the M2-S31N mutation conferring amantadine-resistance. In addition, an S31D mutation in the M2-protein, conferring a similar level of resistance as the S31N mutation, was detected in three isolates obtained in Japan during the 2014/2015 season. None of the isolates possessed the NA-H274Y mutation conferring oseltamivir-resistance, though a few isolates were found to contain mutations at the catalytic residue 151 (D151A/G/N or V) of the NA protein. These variations did not alter the susceptibility to neuraminidase inhibitors and were not detected in the original clinical specimens, suggesting that they had been acquired during their passage in MDCK cells. Novel polymorphisms were detected in the PB1-F2 open-reading frame resulting in truncations in the protein of 24–34 aminoacids in length. Thus, this study has demonstrated the utility of monitoring the full genome of influenza viruses to allow the detection of the potentially fittest lineages. This enhances our ability to predict the strain(s) most likely to persist into the following seasons and predict the potential degree of vaccine match or mismatch with the seasonal influenza season for that year. This will enable the public health and clinical teams to prepare for any related healthcare burden, depending on whether the vaccine match is predicted to be good or poor for that season.

## Introduction

Influenza A viruses are pleiomorphic, lipid-enveloped viruses belonging to the family *Orthomyxoviridae*. It has a single-stranded, segmented, negative-sense RNA genome of ~14 kbp (Webster et al., 1992), which is characterized by a high mutation rate (Suárez et al., 1992; Nobusawa and Sato, 2006). This drives its evolution and adaptation in response to various host and environmental selection pressures. In addition, the segmented genome facilitates the occasional reassortment of genes between different influenza A viruses, leading to the development of antigenically new viruses with pandemic potential (Steel and Lowen, 2014). Some of these reassortment events are detrimental, i.e., they reduce the viral fitness to such a degree that it leads to the disappearance of the reassorted viral population. Alternatively, such events could provide the virus with one or more homotypic (same subtype) or heterotypic (different subtype) genome segments that might boost its infectivity and/or pathogenicity, enabling it to transmit efficiently and to replace older strains (Li and Chen, 2014), as well as facilitating vaccine escape.

Seasonal outbreaks are driven by antigenic drift, which allows the virus to escape host immunologic memory to previous infection- and/or vaccine-induced immunity. In temperate zones, influenza A viruses cause annual winter outbreaks in humans resulting in significant public health and economic burden (Stöhr, 2002). In tropical zones influenza outbreaks occur throughout the year, often with activity peaking during the rainy season (Stephenson and Zambon, 2002).

Occasional antigenic shifts can arise which significantly alters virus antigenicity, leading to pandemics (Scholtissek, 1995). The most recent influenza pandemic was caused by a swine-origin reassortant H1N1 virus in 2009 (H1N1pdm09; Massingale, 2009). This caused over 60 million cases (20% of the population) in the United States alone, with an estimated 274,304 hospitalizations and 12,469 deaths during its first year (Shrestha et al., 2011). This burden was even higher in developing countries, due to a more delayed response and a more resource-limited healthcare infrastructure (Charu et al., 2011). The global deaths attributed to respiratory or cardiovascular complications due to H1N1pmd09 infections have been estimated to be in the range of 151,700–575,400 people (Dawood et al., 2012).

Influenza reassortment events are usually identified by drawing phylogenetic trees of each gene segment and identifying clade jumping events, i.e., clustering of certain strains or isolates in different clades on different gene trees (Steel and Lowen, 2014). Nonetheless, reassortment events among homogenous or very closely related samples are more difficult to detect as these strains tend to cluster together. Genomic reassortment events have been implicated in the emergence of pandemic influenza strains (e.g., the H1N1pdm09; Massingale, 2009), antiviral drug resistance (e.g., the appearance of the M2-S31N seasonal H3N2 amantadine-resistant strain and the NA-H274Y seasonal H1N1 oseltamivir-resistant influenza strain; Simonsen et al., 2007; Zaraket et al., 2010a,b), and novel avian influenza viruses with pandemic potential (e.g., H5N1 and H7N9; Li et al., 2004; Wu et al., 2013).

In this study, we analyzed the extent of interseasonal reassortment events among H3N2 influenza viruses and assessed their potential contribution to the evolution of seasonal strains, using 100 full genome sequences obtained by next generation sequencing of isolates obtained from Japan, Myanmar, Vietnam, and Lebanon. We show evidence of frequent interseasonal reassortment events among H3N2 influenza viruses and report on novel PB1-F2 and M2 gene polymorphisms that confer resistance to amantadine.

## Materials and methods

### Sample collection and ethical approval

Nasopharyngeal swabs were collected from patients with influenza-like illness (ILI) with at least one of the following symptoms: fever >38°C, coughing, rhinorrhea, myalgia, arthralgia, or diarrhea.

In Lebanon, Japan, and Myanmar specimens were collected as part of an influenza surveillance project run by our group. Sample collection in these countries was approved by the ethical committee at the home institution of each contributing laboratory. A written informed consent was obtained from subjects prior to enrollment in the study.

In Vietnam, sample collection was performed as part of Ministry of Health surveillance program for ILI and severe acute respiratory infection (SARI). The National Institute of Hygiene and Epidemiology (NIHE), Vietnam, provided ethical committee approval for the study and all subjects provided written, informed consent.

### Sample selection and virus propagation

One hundred human influenza A/H3N2 clinical isolates collected during July 2012–January 2015 were randomly selected from a bank of isolates available in our laboratory through the aforementioned surveillance programs. All isolates were passaged on Madin–Darby canine kidney (MDCK) cells maintained in minimum essential medium (MEM) supplemented with 10% fetal bovine serum (Gibco).

### Full genome sequencing

Viral RNA was extracted using Extragen II kit (Kainos) as per manufacturer's instructions. Full genome sequencing of H3N2 isolates was performed using next generation sequencing method as previously described (Kanehira et al., 2015).

Briefly, a cDNA library was prepared using random hexamers and NEBNext Ultra™ RNA Library Prep kit (NEB) according to the manufacturer's instructions. This was sequenced using a Miseq second-generation sequencer (Illumina) with Reagent Kit v2 and v3 (Illumina). The genomic sequence of isolates were determined using CLC Genomics Workbench 7.0.4 (CLC bio, Inc.). Output reads from the sequencer were trimmed with a quality score limit of 0.05 and mapped to influenza A/H3N2 virus reference sequence sets with the following settings: mismatch cost = 2, insertion cost = 3, deletion cost = 3, length fraction = 0.5, similarity fraction = 0.8. The accession number of the reference sequence sets used for mapping the full H3N2 genomes are: KF432080, KF014949, JX437916, KF014151, KF014594, KF451895, JX437856, and KF014664. Reads mapping to the dog (source of MDCK cells) reference genome data (Dog, CanFam3.1) available at the University Of California Santa Cruz Genome Browser (http://genome.ucsc.edu/) were subtracted.

Whole genomic sequences were established as consensus sequences from reads mapped to the reference sequences. The mapping of NA and M2 gene were subjected to CLC quality-based variant detection with: minimum coverage = 10 reads; minimum frequency = 10%; neighborhood radius = 5; maximum gap and mismatch count = 2; minimum neighborhood quality = 15; minimum central quality = 20. Amino acid changes conferring resistance to influenza antiviral drugs were analyzed by the CLC amino acid changes tool.

Sanger sequencing was used to confirm the presence of any key mutations in the M2 or NA genes in RNA extracted directly from the original clinical specimens, when these were available. Briefly, cDNA was first performed using universal Uni12 primers for influenza A (Hoffmann et al., 2001) followed by PCR with M2 or NA specific primers (Masuda et al., 2000; Dapat et al., 2009). The PCR products were then purified and sequenced using Big Dye Terminator on an ABI Prism 3130XL Genetic analyzer (Applied Biosystems). Sanger sequences from original samples were not incorporated in the full genome sequences obtained by NGS. All sequences were deposited in the public databases. The accession numbers and data for the isolates are listed in Supplementary Table 1.

### Phylogenetic analysis

Individual gene segments of isolates from this study were aligned with vaccine-strains obtained from the Influenza Resource Database (http://www.ncbi.nlm.nih.gov/genomes/FLU) using CLUSTAL W alignment tool in the BIOEDIT software (Hall, 1999). Maximum likelihood (ML) phylogenies were inferred on the basis of the best fit nucleotide substitution model for each gene as implemented in MEGA 6.0 (Tamura et al., 2013).

The Hasegawa-Kishino-Yano model with a gamma distribution (HKY+G) was used for the PB2, PB1, PA, HA, and NP, the Tamura-Nei model with gamma distribution (T92+G) for the NA, the Kimura 2-parameter for MP, and the T92 for NS.

Initial trees for the heuristic search were obtained by applying the Neighbor-Joining method to a matrix of pairwise distances estimated using the Maximum Composite Likelihood (MCL) approach. A non-parametric bootstrap sampling analysis with 1000 replicates of the ML tree was applied using the best nucleotide substitution model. Clades were designated based on the clustering of isolates in the HA phylogeny with bootstrap support ≥70. Reassortment events were detected by mapping the topologies of viruses across all trees using TreeDyn (http://www.treedyn.org/).

### Antiviral drug susceptibility testing

Phenotypic antiviral drug susceptibility of influenza isolates to neuraminidase inhibitors (NAIs) was assessed by measuring the 50% inhibitory concentrations (IC_50_) of oseltamivir (Sequoia Research, UK), zanamivir (Sequoia Research, UK), peramivir (Shionogi Co., Japan), and laninamivir (Daiichi Sankyo Co., Japan), using a fluorescence-based NA inhibition assay with methylumbelliferone N-acetylneuraminic acid (MUNANA) as the substrate (Hurt et al., 2009; Dapat et al., 2013).

Briefly, each virus isolate was first titrated to obtain a dilution in the linear range of the NA activity curve. NAI assay was performed by adding 25 μL of each NAI dilution (range 0.02–1250 nM) to all wells of a microtiter plate. The virus was diluted to 25,000 fluorescence unit and 25 μL of each dilution was added to all wells. Plates were incubated at 37°C for 30 min. MUNANA substrate (50 μL at a final concentration of 25 μM) was added to each well then the plates were incubated at 37°C for 60 min. The reaction was finally stopped by adding 260 μL of 200 mM of sodium carbonate to each well. Fluorescence was measured using a TriStar LB 941 multi-well plate reader (Berthold Technologies GmbH & Co.).

Amantadine susceptibility assay was performed to determine the effect of the M2 gene mutation on inhibitory activity of amantadine against H3N2 isolates (Masuda et al., 2000). Confluent monolayers of MDCK cells in the 96-well plates were inoculated in triplicate with 0.1 ml of each virus dilution. Maintenance media (0.1 ml) containing 0.2 mg/ml TPCK trypsin was added to each well and the plates were incubated at 37°C in a CO_2_ incubator, where a cytopathic effect was observed at 48 h. The titers were calculated by the Reed–Muench method (Reed and Muench, 1938). To assess the amantadine susceptibility of the H3N2 viruses, the values of the TCID_50_/0.1 ml were compared. A difference of 2.0 log_10_ TCID_50_/0.1 ml or more in the presence and absence of amantadine was an indication of the virus susceptibility to amantadine.

## Results

### HA gene analysis

**Figure 1:**
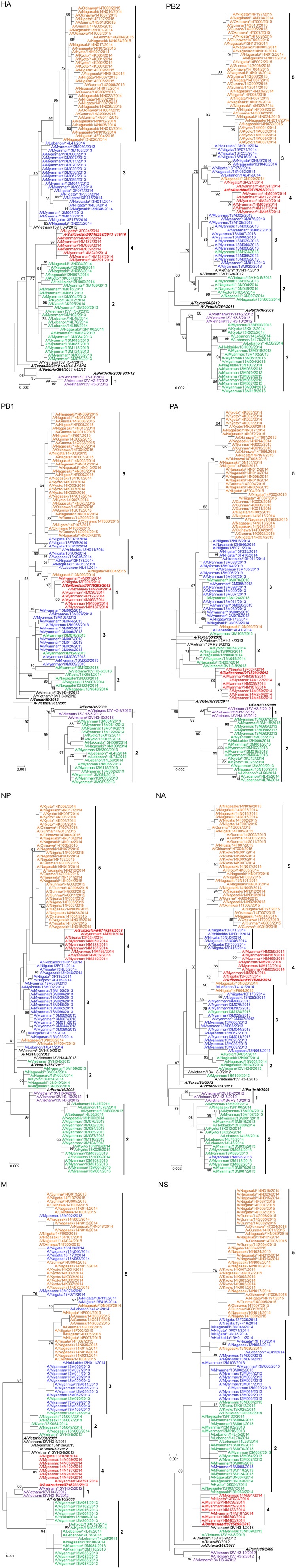
**Evolutionary relationship among human influenza A/H3N2 isolates from Asian countries**. Full genome sequences of 100 H3N2 isolates were aligned and the phylogenetic tree for each genome segment was inferred using maximum likelihood analysis based on the best-fit nucleotide substitution model for each gene. Bootstrap support values ≥70%, which corresponds to a ≥95% probability that a given clade is real, are shown (Hillis and Bull, 1993). Full genome sequences of WHO-recommended vaccine strains for the seasons covered by the study were obtained from the Influenza Resource Database and included in the analysis for comparison. The vaccine strains are indicated in boldface italics. The season(s) covered by the vaccine strains are indicated next to the vaccine strain name in the HA tree: e.g., V11/12 for season 2011/2012, V12/13 for 2012/2013, V13/14 for 2013/2014, V14/15 for 2014/2015, and V15/16 for 2015/2016.

In order to determine the relationship among the H3N2 isolates included in the study and in relation to the vaccine strains, we first inferred the phylogenetic tree of the HA gene using the full coding sequence. A total of 100 isolates collected during 2012–2015 from Japan, Lebanon, Myanmar, and Vietnam were sequenced using the Illumina Miseq platform and included in the analysis. The WHO-recommended vaccine strains for seasons 2011/2012–2015/2016 were also included in the analysis for the purpose of comparison. We arbitrary classified the isolates in five clusters based on the HA tree clustering (Figure 1). All clusters had bootstrap support greater than 85%.

Cluster 1 was formed of Vietnamese isolates collected in 2012 and was closely related to A/Perth/16/2009, the 2011/2012 season vaccine strain. Cluster 2 included isolates collected from the four countries included in the study during the 2013 and 2013/2014 seasons. Cluster 3 contained isolates from Myanmar, Japan, and Lebanon which were collected in the 2013 and 2013/2014 seasons. Cluster 4 consisted mainly of Myanmar isolates and one Japanese isolate that were collected in 2014, which also included the WHO-recommended vaccine strain for the 2015/2016 season (A/Switzerland/9715293/2013). Cluster 5 was composed of the Japanese isolates collected during the 2014/2015 season. It was further noted that clusters 2, 3, 4, and 5 were descended from the A/Texas/50/2012 vaccine strain for the 2013/2014 and 2014/2015 seasons. Overall, the HA tree revealed both a temporal and geographical clustering of samples.

Strains isolated in 2013/2014 in the northern hemisphere (Lebanon and Japan) seemed to originate from those that had been circulating several months earlier in tropical Myanmar during 2013. Multiple lineages were observed, co-circulating in different countries, as in the case of clusters 2 and 3 during the 2013 (tropical) and 2013/2014 (temperate) influenza seasons. Two isolates collected in Vietnam, A/Vietnam/13V H3-9/2012 and A/Vietnam/13V H3-4/2013, could not be assigned to any of the clusters and were designated as singleton intermediates of major clusters. This small number of Vietnamese isolates may have hindered our ability to accurately infer their relationship with strains that were circulating in other countries.

### Full genome analysis

In order to investigate the full genome evolution of the circulating strains, we next analyzed the phylogenetic tree of each segment (Figure 1).

Cluster 2 isolates of the HA tree were found in two distinct clusters in the phylogenies of the seven other genes. One cluster was exclusively formed of closely related Myanmar isolates, while the rest of the isolates from Japan and Lebanon seemed to be more distantly related despite clustering together. This observation suggests that cluster 2 samples evolved into two lineages sharing the same HA gene as a result of 1+7 reassortment event.

Cluster 3 isolates consistently grouped together in the trees of PA, HA NP, and NS but divided into two distinct clusters in the PB2, PB1, and NA trees. In the M tree, a subset of cluster 3 isolates intermingled among those of cluster 5 in close proximity to the rest of the isolates from cluster 3. This suggests that cluster 3 emerged into two lineages as a result of a 5+3 reassortment event.

Cluster 4 isolates consistently grouped together but possessed different topologies in different gene trees. This cluster, which is mainly formed of Myanmar isolates, was closely related to cluster 2 in the HA, PA, M, and NS tree but it associated with cluster 3 in the PB2, PB1, NP, and NA trees indicating a 4+4 reassortment event involving a cluster 2 descendent strain with a cluster 3 derived strain.

Several other singleton reassortants were also identified (summarized in Table 1). A common feature of these isolates is that they all shared a common ancestral gene PB1 gene, which belonged to cluster 3. Thus, PB1 gene of cluster 3 may have a fitness advantage over other lineages, allowing it to persist into subsequent seasons.

**Table 1:** **Genetic makeup of the singleton reassortant isolates detected in this study**.

**Sample ID**	**Gene segment cluster**							
	**PB2**	**PB1**	**PA**	**HA**	**NP**	**NA**	**M**	**NS**
A/Myanmar/13M070/2013	2	3	3	2	2	3	3	2
A/Myanmar/13M098/2013	3	3	3	3	3	2	3	2
A/Myanmar/13M124/2013	3	3	3	2	2	2	3	2
A/Nagasaki/13N020/2014	3	3	3	5	3	3	5	3
A/Niigata/14F004/2015	5	3	5	5	3	3	5	5

### Antiviral drug resistance markers

In order to determine the genotypic antiviral drug susceptibility of the H3N2 isolates included in this study we analyzed the M2 and NA protein coding sequences for the reported genetic markers of resistance against M2-channel blockers (amantadine and rimantadine) and neuraminidase inhibitors (NAIs; oseltamivir, zanamivir, laninamivir, and peramivir). Five mutations (L26F, V27A, A30T, S31N, and G34E) in the transmembrane region of the M2-channel protein of influenza A virus have been reported to confer resistance to M2-channel blockers. Mutations E119V/I, Q136K, D151A/D, I222V, R292K, and N294S in the NA protein have been reported to cause reduced susceptibility to NAIs (Abed et al., 2006; Dapat et al., 2010; Mishin et al., 2014). In this study, 60/63 samples possessed the S31N mutation conferring resistance to M2-channel blockers. The remaining three isolates (A/Nagasaki/14N010/2014, A/Nagasaki/14N012/2014, and A/Nagasaki/14N013/2014) had an S31D mutation. This mutation was also detected in the original clinical samples, using Sanger sequencing (Table 2). These three isolates were identified during the 2014/2015 seasons in Japan and formed a subgroup within cluster 5.

**Table 2:** **Amantadine susceptibility of H3N2 isolates with S31D M2 mutation**.

**Sample ID**	**Amino acid at residue 31^a^**	**Log_10_TCID_50_/0.1 ml**			**Phenotype**
		**Am(–)^b^**	**Am (+)^c^**	**Difference**	
A/Nagasaki/14N010/2014	D	4.5	4.8	0.3	Resistant
A/Nagasaki/14N012/2014	D^d^	1.5	1.5	0.0	Resistant
A/Nagasaki/14N013/2014	D^d^	3.5	3.5	0.0	Resistance
A/Nagasaki/05N230/2006	S	4.8	2.8	2.0	Sensitive
A/Okinawa/14T006/2015	N	3.5	3.5	0.0	Resistant

a *Data using NGS of MDCK-passaged samples*.

b *Amantadine absent*.

c *Amantadine present*.

d *The mutation was also confirmed in the original sample using Sanger sequencing*.

Phenotypic amantadine susceptibility assay, revealed that the S31D mutation confers a similar level of resistance as the widely spread S31N mutation, as demonstrated by the similar titers in presence and absence of amantadine (Table 2). In contrast, the reference amantadine-susceptible S31 strain demonstrated a difference of 2 log_10_ – TCID_50_/0.1 ml in presence and absence of amantadine.

No variations were identified in any of the known NAI resistance markers except for residue 151 of the NA enzyme catalytic site for which several variants were detected by deep sequencing analysis. Variants (A, G, N, or V) at residue 151 were co-detected with the wild-type (D) with an average variant frequency of 48% (range 7.7–84.7%).

One isolate possessed a D151A mutation, two had a D151V mutation, 10 possessed a D151G mutation, and 27 had a D151N mutation. In order to assess the effect of these mutations on the susceptibility of the isolates to NAIs, we performed IC_50_ analysis of 11 representative isolates with substitutions D151G/A/or V. All of the samples had IC_50_-values corresponding to the reference susceptible strain (Table 3). Sanger sequencing of the original clinical samples of these isolates showed clear single peaks for the D codon at residue 151 (Table 3). Therefore, these mutations might have emerged in cell culture, and their presence as a mixed population with the D151 does not alter the IC_50_ of NAIs.

**Table 3:** **IC_**50**_ for NAIs against influenza samples with amino acid variation at NA 151 residue**.

**Sample ID**	**Residue 151 (N2 numbering)**		**IC_50_ (nM)**			
	**Sanger^a^**	**NGS^b^**	**Oseltamivir**	**Zanamivir**	**Peramivir**	**Laninamivir**
A/Niigata/13F335/2014	D	G	0.72	0.47	0.18	0.68
A/Niigata/13F416/2014	D	G	0.65	0.44	0.16	0.60
A/Okinawa/14T003/2015	D	G	0.15	0.94	0.50	0.60
A/Myanmar/13M004/2013	D	G	0.71	1.05	0.10	0.64
A/Myanmar/13M006/2013	D	V	0.63	0.53	0.10	0.45
A/Myanmar/13M007/2013	D	G	0.69	0.59	0.13	0.48
A/Myanmar/13M035/2013	D	G	0.61	1.07	0.12	0.68
A/Myanmar/13M084/2013	D	V	0.59	0.85	0.08	0.66
A/Myanmar/13M102/2013	D	G	0.64	0.98	0.12	0.59
A/Myanmar/13M109/2013	D	A	0.88	0.67	0.13	0.51
A/Myanmar/13M300/2013	D	G	0.47	1.21	0.10	0.68
A/FUKUI/20/2004^c^	D	-	0.50	1.26	0.14	0.93

a *Sanger sequence result of original clinical specimen*.

b *Next generation sequence result of MDCK-passaged samples*.

c *Reference strain*.

### Novel PB1-F2 polymorphisms

The PB1-F2 protein is a non-structural protein encoded by a +1 alternate open reading frame (ORF) in the PB1 gene segment (Chen et al., 2001). By analyzing the PB1-F2 ORF, we found five isolates with polymorphisms leading to an early stop codon resulting in a truncated protein. A/Niigata/13F416/2014 and A/Niigata/13F335/2014 of cluster 3 had a CGA to TGA polymorphism leading to PB1-F2 truncated protein of 24 amino acids (aa). A/Vietnam/13V H3-2/2012 of cluster 1 had CAA to TAA polymorphism leading to a 25 aa PB1-F2 protein.

Three Lebanese isolates, A/Lebanon/14L45/2014, A/Lebanon/14L56/2014, and A/Lebanon/14L78/2014 belonging to cluster 2 had a TCA to TAA polymorphism resulting in a truncated PB1-F2 protein with 34 aa. These three isolates clustered together in all segment phylogenies; two were identical and one had slightly diversified relative to the two other strains. This truncation appeared to have no detrimental effect on the infectivity and transmissibility of these viruses.

**Figure 2:**
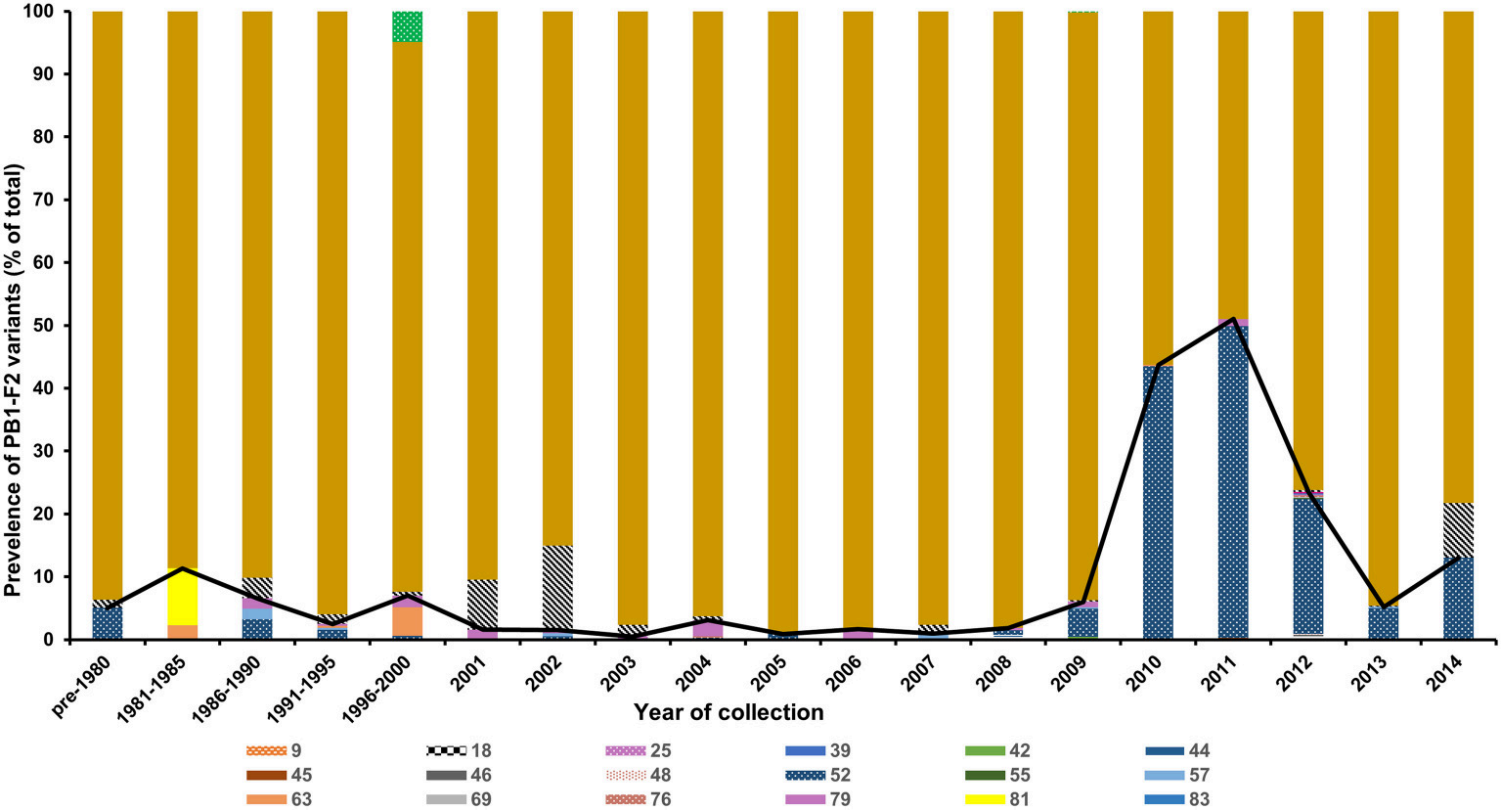
**Frequency of PB1-F2 polymorphisms leading to a truncated protein**. The PB1-F2 coding region of 5365 H3N2 isolates reported between 1968 and 2014 at the Influenza Virus Resource were analyzed to determine the polymorphisms leading to an early stop codon in the PB1-F2 open reading frame. The predicted protein/peptide length in amino acid is indicated in the figure.

We next assessed the historic global prevalence of these polymorphisms by analyzing all the human H3N2 PB1 gene sequences available in the Influenza Virus Resource (http://www.ncbi.nlm.nih.gov/genomes/FLU/FLU.html). Analysis of PB1-F2 coding sequence of 5365 deposited PB1 sequences revealed that the full-length PB1-F2 of 87 or 90 aa constituted the majority of reported sequences (87.9%; Figure 2). PB1-F2 of 52 aa was the most prevalent truncation accounting for 10% of all samples. The prevalence of this polymorphism rapidly surged during 2010–2012, constituting 22–50% of all reported sequences during these years but dropped again in frequency in the subsequent years. Overall, 19 different truncations were reported in the database, including one (25 aa) similar in length to the truncation detected in the Vietnamese isolate in this study.

## Discussion

Influenza A/H3N2 viruses have been shown to exhibit the fastest evolutionary rate compared to other contemporary human influenza viruses such as seasonal H1N1 and influenza B (Nobusawa and Sato, 2006; Zaraket et al., 2009). This difference is attributable to the higher fixation rate of mutations acting upon H3N2 viruses (Zaraket et al., 2009). Reassortment events among multiple lineages can aid in the fixation of mutations in the genome if they enhance viral fitness. These events might also force further selection of additional mutations in the genome to retain the functional balance among the virus components (Li and Chen, 2014; Steel and Lowen, 2014).

In this study, we describe the full genome analysis of 100 influenza A/H3N2 isolates obtained during 2012–2015 from four Asian countries: Japan, Lebanon, Myanmar, and Vietnam. Our analysis revealed the co-circulation of multiple lineages of H3N2 strains during the same period sharing the same ancestry of the HA protein. We also report the emergence of a rare amantadine-resistance conferring mutation, S31D, and several novel PB1-F2 polymorphisms. Several reassortment events among H3N2 isolates were also detected especially during the 2013 (tropical) and 2013/2014 (temperate) seasons, during which four major lineages sharing the same ancestry co-circulated along with other minor lineages or singleton strains. This phenomena has been previously described for H3N2 viruses in past influenza seasons (Holmes et al., 2005; Nelson and Holmes, 2007). Some of the lineages detected in this study, e.g., lineages belonging to clusters 2 or 3, shared the same HA protein as evident by the close clustering of these isolates in the HA tree.

### HA gene

Interestingly, despite a change in the composition of the other genes, the HA protein appears to have undergone a parallel evolution, likely under similar selection pressures as those exerted on these closely related lineages. In contrast to the 2013 and 2013/2014 strains, most of the 2014/2015 season isolates belonged to one lineage and sporadic singleton reassortant strains were observed.

Notably, the 2014/2015 strains (cluster 5) emerged from cluster 3 rather than cluster 2, which circulated in the previous season. Also the cluster 3 PB1 gene was shared by all of the singleton reassortants observed in our study. This suggests a fitness advantage of the gene constellation of lineage 3 enabling it to persist into the following season. Currently, the selection of vaccine strains for each season is based on the degree of antigenic drift and the prediction of which strain might predominate in the following season (Stöhr et al., 2012). Our data as well as others highlight the importance of considering full genome sequences, in addition to antigenic data, to predict the influenza strain that is most likely to prevail in the next influenza season (Belanov et al., 2015).

Additionally, we noticed that strains circulating in Japan (northern Hemisphere) originated from those circulating months earlier in the tropical region, as shown by the co-clustering of Japanese and Myanmar isolates from successive seasons—particularly in the HA tree. These findings are in agreement with our previous data showing that amantadine-resistant H3N2 influenza strains circulated in the subtropical islands of Okinawa several months before their detection in the main islands of Japan (Suzuki et al., 2009). This data supports the monitoring of influenza in tropical regions to improve the selection process of vaccine strains. Furthermore, Japan uses more influenza antivirals than anywhere else in the world subjecting viruses circulating in this country to this specific selection pressure (Monto, 2009). This increases the likelihood for emergence of resistant strains, indicating the need for comprehensive influenza sequence surveillance in this country.

### PB1 gene

The PB1-F2 is a pro-apoptotic protein that is localized to the mitochondria (Chen et al., 2001). Other roles for PB1-F2 like the promotion of inflammation and the regulation of viral polymerase activity have been also described (McAuley et al., 2007; Mazur et al., 2008). Nonetheless, its contribution to virulence remains controversial and is likely to be host and strain dependent (McAuley et al., 2010). The full length PB1-F2 is 87–90 aa in length, however, mutations in the ORF could lead to early stop codons resulting in a truncated product.

In this study, all samples had a full length PB1-F2 protein except for six isolates. These isolates possessed truncated PB1-F2 proteins with 24, 25, or 34 aa residues, five of which were novel polymorphisms leading to an early stop codon in the ORF. Three of these isolates, containing a 34 aa-length PB1-F2, were isolated within a 1-month period in Lebanon and clustered closely in all of the gene trees. The ability of these viruses to circulate in the community implies a minimal effect of the PB1-F2 truncation on their transmissibility and infectivity. Analysis of all previously reported human H3N2 viruses revealed that the vast majority of all isolates possessed a complete length PB1-F2 (87 or 90 aa).

The most common variant was a PB1-F2 of 52 aa in length, which accounted for almost 50% of all reported isolates in 2011 and 2012 before sharply dropping again in prevalence in the following years to be replaced by the full length variant. The impact of PB1-F2 truncations on the infectivity and transmissibility of H3N2 viruses is not known. Gibbs et al. showed that PB1-F2 amino acids 69–82 constitute the minimal mitochondrial translocation signal (Gibbs et al., 2003). In contrast, Yamada et al. showed that amino acids 63–75 are the minimal sequence required to allow mitochondrial localization (Yamada et al., 2004). The H1N1pdm09 virus, despite lacking the PB1-F2 protein, was able to widely transmit and become pandemic by establishing in humans. Expression of the full length PB1-F2 by the H1N1pdm09 had a minimal impact on the virus virulence in mice and ferrets (Hai et al., 2010). Studies to better elucidate the effect of PB1-F2 truncations on virulence of seasonal H3N2 viruses are warranted.

### M gene

All of our H3N2 viruses had the M2-S31N mutation conferring resistance to amantadine, except three isolates. These isolates were collected in Nagasaki within a short period during the 2014/2015 season and possessed an S31D mutation. This mutation has been sporadically reported in Japan and Korea in 2006 and 2008 (Hata et al., 2007; Baek et al., 2009).

Baek et al. reported that the S31D mutation did not cause resistance of H3N2 isolates to amantadine contrary to the S31N mutation (Baek et al., 2009), but later demonstrated that this mutation reduces the ability of amantadine to block the M2-channel to a level comparable to that of the S31N mutation (Balannik et al., 2010). Our data confirmed that the S31D mutation causes phenotypic resistance to amantadine at a similar level as the S31N mutation.

The three Nagasaki isolates with the S31D mutation were closely related, clustering together with another S31N containing strain from Nagasaki in all gene trees. The four strains possessed identical PA genes but were slightly divergent in the other genes. Therefore, we speculate that the S31D mutant virus has emerged from an S31N strain by a single-point mutation of AAT(N) to GAT(D). Future monitoring of the S31D mutation among clinical specimens is critical to determine its prevalence and fitness-cost on H3N2 viruses.

### NAI susceptibility

All of the tested isolates were susceptible to the four NAIs tested. A few isolates show some variation at residue 151 (D151A/G/N/ or V) in the catalytic site of the NA enzyme active site. Variations at residue 151 have been previously described as common among influenza isolates (McKimm-Breschkin et al., 2003; Lin et al., 2010). Residue 151 has been suggested as a proton donor in the NA catalytic reaction during the binding of the enzyme to the sialic-acid containing receptor (Varghese et al., 1992) and to induce receptor binding ability of the NA gene (Lin et al., 2010; Mohr et al., 2015). As a result, the receptor binding affinity of the virus increases which could bias the interpretation of hemagglutination inhibition data used by the WHO for vaccine selection (Lee et al., 2013).

Changes at this residue also affect the enzymatic activity. The D151E possesses <10% of the wild-type enzyme activity (McKimm-Breschkin et al., 2003), resulting in a 10-fold increase in the IC_50_ of oseltamivir (Yen et al., 2006). Additionally, D151A and D151G variations were shown to cause a 30- and 1000-fold increase, respectively, in zanamivir IC_50_s compared to D151 (Sheu et al., 2008; Mishin et al., 2014). However, in this study, isolates possessing variations at residue 151 had IC_50_s comparable to the reference strain containing the wild-type D151 residue. These variations were present at an average frequency of 48% along with the wild-type residue, which could have masked their effect on NA activity and susceptibility to NAIs.

These findings should be taken with care, as the D151G/N/A substitutions have not been reported in patients treated with NAIs and are likely to be an artifact of passaging H3N2 virus in MDCK cells (Sheu et al., 2008; Lee et al., 2013; Mishin et al., 2014). Studies reporting these mutations used viruses isolated in MDCK cells (McKimm-Breschkin et al., 2003; Lin et al., 2010). Consistently, sequencing of the original (uncultured) clinical samples in our study revealed that, unlike the MDCK-passaged isolated, they all possessed the wildtype D151 residue. This confirms that variations at this residue are very likely cell culture-induced. Therefore, when reporting new NA resistant variants it is essential to confirm the results in the original clinical samples and not only using isolates.

One limitation of the study is that NGS was performed on MDCK-isolates rather than original samples. While this could introduce some artifacts to the data such as cell-induced mutations (e.g., residue 151 variants), it is unlikely to affect our analysis of reassortment events. To overcome this limitation, we also confirmed key M2 and NA mutations in the original samples using Sanger sequencing.

## Conclusion

In conclusion, the frequency of reassortment events detected in this study and the observation that one out of several genetic constellations that circulate in a given season might seed subsequent epidemics has important implications for selection of vaccine strains. Choosing a vaccine strain that not only matches the antigenically drifted strain from a previous season but also one that possess a better fit constellation is likely to improve the success of vaccine-strain selection. Finally, even though amantadine is considered an obsolete medication due to the universal carriage of S31N-M2 mutation among contemporary H3N2 isolates, the detection of an S31D mutation suggests that this residue is still undergoing mutations with the potential to revert back to the susceptible S31 genotype.

## Japanese influenza collaborative study group

**Shinji Kimura**, Matsumae Town Hospital, Hokkaido, Japan; **Takashi Kawashima**, Kawashima Internal Medicine Clinic, Gunma, Japan; **Isamu Sato**, Yoiko Pediatric Clinic, Niigata, Japan; **Shigeyoshi Hibi**, Hibi Clinic for Children, Kyoto, Japan; **Naoki Kodo**, Kodo Children's Clinic, Kyoto, Japan; **Hironori Masaki**, Masaki Respiratory Medicine Clinic, Nagasaki, Japan; **Yutaka Shirahige**, Shirahige Internal Medicine Clinic, Nagasaki, Japan; **Norichika Asoh**, Juzenkai Hospital, Nagasaki, Japan; **Yoshiko Kita**, Juzenkai Hospital, Nagasaki, Japan; **Reiki Kuroki**, Tagami Hospital, Nagasaki, Japan; **Yasuo Nawata**, Tagami Hospital, Nagasaki, Japan; **Yasuhiko Ono**, Ono Pediatric Clinic, Nagasaki, Japan; **Tomoko Makiya**, Department of Pediatrics, Okinawa Chubu Hospital, Okinawa, Japan; **Kiyotaka Takefuta**, Department of Pediatrics, Okinawa Chubu Hospital, Okinawa, Japan.

## Author contributions

HZ and RS designed the study, performed the phylogentic analysis, and drafted the manuscript. HK, AH, RY, TO, NT, RT, TS, and MH helped design the study, performed experiments, and helped with the analysis and drafting the manuscript. JT helped with the analysis and drafting the manuscript. The Japanese study group, YM, YK, KO, HT, NL, NA, NH, LM, and GD helped with the study design, sample collection, and data analysis, and manuscript. YS helped with the study design, data analysis, and manuscript.

### Conflict of interest statement

The authors declare that the research was conducted in the absence of any commercial or financial relationships that could be construed as a potential conflict of interest.
