# Supplementary material for: Full Genome Characterization of Human Influenza A/H3N2 Isolates from Asian Countries Reveals a Rare Amantadine Resistance-Conferring Mutation and Novel PB1-F2 Polymorphisms
Source: Front Microbiol. 2016 Mar 7;7:262. doi: 10.3389/fmicb.2016.00262 (PMC4779883; doi:10.3389/fmicb.2016.00262)
Supplement: Supplementary file 1 [file Table1.PDF]

Supplementary Table 1. Accession numbers of the NGS data and consensus sequences of the eight genome segments of H3N2 isolates

Sample ID	Country	Season	Collection date dd/mm/yyyy	Database		Consensus sequence accession number								NGS data accession number DDBJ
				DDBJ	GISAID	PB2	PB1	PA	HA	NP	NA	M	NS	
A/Kyoto/13K012/2014	Japan	2013-2014	24/01/2014	○		LC032878	LC032879	LC032880	LC032881	LC032882	LC032883	LC032884	LC032885	DRR051388
A/Kyoto/13K025/2014	Japan	2013-2014	05/02/2014	○		LC032870	LC032871	LC032872	LC032873	LC032874	LC032875	LC032876	LC032877	DRR051389
A/Kyoto/13K054/2014	Japan	2013-2014	13/02/2014	○		LC032886	LC032887	LC032888	LC032889	LC032890	LC032891	LC032892	LC032893	DRR051390
A/Hokkaido/13H009/2014	Japan	2013-2014	14/01/2014	○		LC032990	LC032991	LC032992	LC032993	LC032994	LC032995	LC032996	LC032997	DRR051386
A/Hokkaido/13H011/2014	Japan	2013-2014	22/01/2014	○		LC032998	LC032999	LC033000	LC033001	LC033002	LC033003	LC033004	LC033005	DRR051387
A/Niigata/13NU003/2014	Japan	2013-2014	30/01/2014	○		LC033054	LC033055	LC033056	LC033057	LC033058	LC033059	LC033060	LC033061	DRR051461
A/Niigata/13F173/2014	Japan	2013-2014	17/02/2014	○		LC033110	LC033111	LC033112	LC033113	LC033114	LC033115	LC033116	LC033117	DRR051458
A/Niigata/13F071/2014	Japan	2013-2014	05/02/2014	○		LC033118	LC033119	LC033120	LC033121	LC033122	LC033123	LC033124	LC033125	DRR051457
A/Niigata/13F024/2014	Japan	2013-2014	31/01/2014	○		LC033126	LC033127	LC033128	LC033129	LC033130	LC033131	LC033132	LC033133	DRR051456
A/Niigata/13F335/2014	Japan	2013-2014	03/03/2014	○		LC033134	LC033135	LC033136	LC033137	LC033138	LC033139	LC033140	LC033141	DRR051459
A/Niigata/13F416/2014	Japan	2013-2014	10/03/2014	○		LC033142	LC033143	LC033144	LC033145	LC033146	LC033147	LC033148	LC033149	DRR051460
A/Nagasaki/13N004/2014	Japan	2013-2014	02/01/2014	○		LC033302	LC033303	LC033304	LC033305	LC033306	LC033307	LC033308	LC033309	DRR051435
A/Nagasaki/13N007/2014	Japan	2013-2014	13/01/2014	○		LC033310	LC033311	LC033312	LC033313	LC033314	LC033315	LC033316	LC033317	DRR051436
A/Nagasaki/13N020/2014	Japan	2013-2014	23/01/2014	○		LC033318	LC033319	LC033320	LC033321	LC033322	LC033323	LC033324	LC033325	DRR051437
A/Nagasaki/13N046/2014	Japan	2013-2014	30/01/2014	○		LC033326	LC033327	LC033328	LC033329	LC033330	LC033331	LC033332	LC033333	DRR051438
A/Nagasaki/13N049/2014	Japan	2013-2014	03/02/2014	○		LC033334	LC033335	LC033336	LC033337	LC033338	LC033339	LC033340	LC033341	DRR051439
A/Nagasaki/13N053/2014	Japan	2013-2014	20/02/2014		○	EPI559435	EPI559436	EPI559437	EPI559438	EPI559439	EPI559440	EPI559441	EPI559442	DRR051440
A/Nagasaki/13N063/2014	Japan	2013-2014	03/03/2014		○	EPI562387	EPI562403	EPI562380	EPI562376	EPI562394	EPI562379	EPI562377	EPI562378	DRR051441
A/Nagasaki/13N100/2014	Japan	2013-2014	29/03/2014		○	EPI566929	EPI566931	EPI566928	EPI566924	EPI566930	EPI566927	EPI566925	EPI566926	DRR051442
A/Nagasaki/13N101/2014	Japan	2013-2014	29/07/2014		○	EPI566937	EPI566939	EPI566936	EPI566932	EPI566938	EPI566935	EPI566933	EPI566934	DRR051443
A/Okinawa/14T003/2015	Japan	2014-2015	06/01/2015		○	EPI580590	EPI580592	EPI580589	EPI580585	EPI580591	EPI580588	EPI580586	EPI580587	DRR051454
A/Okinawa/14T004/2015	Japan	2014-2015	13/01/2015		○	EPI580598	EPI580600	EPI580597	EPI580593	EPI580599	EPI580596	EPI580594	EPI580595	DRR051455
A/Okinawa/14T006/2015	Japan	2014-2015	20/01/2015		○	EPI580606	EPI580608	EPI580605	EPI580601	EPI580607	EPI580604	EPI580602	EPI580603	DRR051469
A/Okinawa/14T007/2015	Japan	2014-2015	22/01/2015		○	EPI580614	EPI580616	EPI580613	EPI580609	EPI580615	EPI580612	EPI580610	EPI580611	DRR051470
A/Kyoto/14K001/2014	Japan	2014-2015	08/12/2014		○	EPI577625	EPI577627	EPI577624	EPI577620	EPI577626	EPI577623	EPI577621	EPI577622	DRR051391
A/Kyoto/14K002/2014	Japan	2014-2015	08/12/2014		○	EPI577633	EPI577635	EPI577632	EPI577628	EPI577634	EPI577631	EPI577629	EPI577630	DRR051392
A/Kyoto/14K003/2014	Japan	2014-2015	09/12/2014		○	EPI577641	EPI577643	EPI577640	EPI577636	EPI577642	EPI577639	EPI577637	EPI577638	DRR051393
A/Kyoto/14K005/2014	Japan	2014-2015	09/12/2014		○	EPI577649	EPI577651	EPI577648	EPI577644	EPI577650	EPI577647	EPI577645	EPI577646	DRR051394
A/Kyoto/14K007/2014	Japan	2014-2015	15/12/2014		○	EPI577657	EPI577659	EPI577656	EPI577652	EPI577658	EPI577655	EPI577653	EPI577654	DRR051395
A/Gunma/14G002/2015	Japan	2014-2015	10/01/2015		○	EPI577665	EPI577667	EPI577664	EPI577660	EPI577666	EPI577663	EPI577661	EPI577662	DRR051379
A/Gunma/14G003/2015	Japan	2014-2015	13/01/2015		○	EPI577673	EPI577675	EPI577672	EPI577668	EPI577674	EPI577671	EPI577669	EPI577670	DRR051380
A/Gunma/14G004/2015	Japan	2014-2015	13/01/2015		○	EPI577681	EPI577683	EPI577680	EPI577676	EPI577682	EPI577679	EPI577677	EPI577678	DRR051381
A/Gunma/14G005/2015	Japan	2014-2015	13/01/2015		○	EPI577689	EPI577691	EPI577688	EPI577684	EPI577690	EPI577687	EPI577685	EPI577686	DRR051382
A/Gunma/14G008/2015	Japan	2014-2015	14/01/2015		○	EPI577697	EPI577699	EPI577696	EPI577692	EPI577698	EPI577695	EPI577693	EPI577694	DRR051383
A/Gunma/14G011/2015	Japan	2014-2015	14/01/2015		○	EPI580550	EPI580552	EPI580549	EPI580545	EPI580551	EPI580548	EPI580546	EPI580547	DRR051384
A/Gunma/14G013/2015	Japan	2014-2015	15/01/2015		○	EPI580558	EPI580560	EPI580557	EPI580553	EPI580559	EPI580556	EPI580554	EPI580555	DRR051385
A/Niigata/14F002/2015	Japan	2014-2015	13/01/2015		○	EPI577580	EPI577581	EPI577582	EPI577583	EPI577584	EPI577585	EPI577586	EPI577587	DRR051462
A/Niigata/14F004/2015	Japan	2014-2015	13/01/2015		○	EPI577593	EPI577595	EPI577592	EPI577588	EPI577594	EPI577591	EPI577589	EPI577590	DRR051463
A/Niigata/14F005/2015	Japan	2014-2015	13/01/2015		○	EPI577601	EPI577603	EPI577600	EPI577596	EPI577602	EPI577599	EPI577597	EPI577598	DRR051464
A/Niigata/14F007/2015	Japan	2014-2015	13/01/2015		○	EPI577609	EPI577611	EPI577608	EPI577604	EPI577610	EPI577607	EPI577605	EPI577606	DRR051465
A/Niigata/14F009/2015	Japan	2014-2015	13/01/2015		○	EPI577617	EPI577619	EPI577616	EPI577612	EPI577618	EPI577615	EPI577613	EPI577614	DRR051466
A/Niigata/14F067/2015	Japan	2014-2015	19/01/2015		○	EPI580526	EPI580528	EPI580525	EPI580521	EPI580527	EPI580524	EPI580522	EPI580523	DRR051467
A/Niigata/14F197/2015	Japan	2014-2015	26/01/2015		○	EPI580534	EPI580536	EPI580533	EPI580529	EPI580535	EPI580532	EPI580530	EPI580531	DRR051468
A/Nagasaki/14N005/2014	Japan	2014-2015	22/09/2014		○	EPI566945	EPI566947	EPI566944	EPI566940	EPI566946	EPI566943	EPI566941	EPI566942	DRR051444
A/Nagasaki/14N010/2014	Japan	2014-2015	04/11/2014		○	EPI577753	EPI577755	EPI577752	EPI577748	EPI577754	EPI577751	EPI577749	EPI577750	DRR051445
A/Nagasaki/14N012/2014	Japan	2014-2015	21/11/2014		○	EPI580622	EPI580624	EPI580621	EPI580617	EPI580623	EPI580620	EPI580618	EPI580619	DRR051471
A/Nagasaki/14N013/2014	Japan	2014-2015	09/12/2014		○	EPI580630	EPI580632	EPI580629	EPI580625	EPI580631	EPI580628	EPI580626	EPI580627	DRR051472
A/Nagasaki/14N014/2014	Japan	2014-2015	12/12/2014		○	EPI577705	EPI577707	EPI577704	EPI577700	EPI577706	EPI577703	EPI577701	EPI577702	DRR051448
A/Nagasaki/14N015/2014	Japan	2014-2015	15/12/2014		○	EPI577713	EPI577715	EPI577712	EPI577708	EPI577714	EPI577711	EPI577709	EPI577710	DRR051449
A/Nagasaki/14N017/2014	Japan	2014-2015	24/12/2014		○	EPI577721	EPI577723	EPI577720	EPI577716	EPI577722	EPI577719	EPI577717	EPI577718	DRR051450
A/Nagasaki/14N018/2014	Japan	2014-2015	25/12/2014		○	EPI577729	EPI577731	EPI577728	EPI577724	EPI577730	EPI577727	EPI577725	EPI577726	DRR051451
A/Nagasaki/14N023/2014	Japan	2014-2015	28/12/2014		○	EPI577737	EPI577739	EPI577736	EPI577732	EPI577738	EPI577735	EPI577733	EPI577734	DRR051452
A/Nagasaki/14N024/2015	Japan	2014-2015	05/01/2015		○	EPI580566	EPI580568	EPI580565	EPI580561	EPI580567	EPI580564	EPI580562	EPI580563	DRR051446
A/Nagasaki/14N039/2015	Japan	2014-2015	14/01/2015		○	EPI580574	EPI580576	EPI580573	EPI580569	EPI580575	EPI580572	EPI580570	EPI580571	DRR051447
A/Nagasaki/14N072/2015	Japan	2014-2015	23/01/2015		○	EPI580582	EPI580584	EPI580581	EPI580577	EPI580583	EPI580580	EPI580578	EPI580579	DRR051453
A/Myanmar/13M002/2013	Myanmar	2013	27/06/2013		○	EPI562627	EPI562629	EPI562626	EPI562622	EPI562628	EPI562625	EPI562623	EPI562624	DRR051400
A/Myanmar/13M004/2013	Myanmar	2013	02/07/2013		○	EPI562635	EPI562637	EPI562634	EPI562630	EPI562636	EPI562633	EPI562631	EPI562632	DRR051401
A/Myanmar/13M006/2013	Myanmar	2013	04/07/2013		○	EPI566199	EPI566201	EPI566198	EPI566194	EPI566200	EPI566197	EPI566195	EPI566196	DRR051402
A/Myanmar/13M007/2013	Myanmar	2013	04/07/2013		○	EPI562643	EPI562645	EPI562642	EPI562638	EPI562644	EPI562641	EPI562639	EPI562640	DRR051403
A/Myanmar/13M011/2013	Myanmar	2013	09/07/2013		○	EPI566207	EPI566209	EPI566206	EPI566202	EPI566208	EPI566205	EPI566203	EPI566204	DRR051404
A/Myanmar/13M016/2013	Myanmar	2013	11/07/2013		○	EPI564844	EPI564846	EPI564843	EPI564839	EPI564845	EPI564842	EPI564840	EPI564841	DRR051405
A/Myanmar/13M020/2013	Myanmar	2013	11/07/2013		○	EPI566215	EPI566217	EPI566214	EPI566210	EPI566216	EPI566213	EPI566211	EPI566212	DRR051406
A/Myanmar/13M029/2013	Myanmar	2013	15/07/2013		○	EPI564852	EPI564854	EPI564851	EPI564847	EPI564853	EPI564850	EPI564848	EPI564849	DRR051407
A/Myanmar/13M035/2013	Myanmar	2013	15/07/2013		○	EPI566223	EPI566225	EPI566222	EPI566218	EPI566224	EPI566221	EPI566219	EPI566220	DRR051408
A/Myanmar/13M044/2013	Myanmar	2013	15/07/2013		○	EPI566532	EPI566534	EPI566531	EPI566527	EPI566533	EPI566530	EPI566528	EPI566529	DRR051409
A/Myanmar/13M056/2013	Myanmar	2013	16/07/2013		○	EPI564860	EPI564862	EPI564859	EPI564855	EPI564861	EPI564858	EPI564856	EPI564857	DRR051410
A/Myanmar/13M061/2013	Myanmar	2013	16/07/2013		○	EPI566540	EPI566542	EPI566539	EPI566535	EPI566541	EPI566538	EPI566536	EPI566537	DRR051411
A/Myanmar/13M062/2013	Myanmar	2013	17/07/2013		○	EPI566548	EPI566550	EPI566547	EPI566543	EPI566549	EPI566546	EPI566544	EPI566545	DRR051412
A/Myanmar/13M070/2013	Myanmar	2013	17/07/2013		○	EPI566620	EPI566622	EPI566619	EPI566615	EPI566621	EPI566618	EPI566616	EPI566617	DRR051413
A/Myanmar/13M076/2013	Myanmar	2013	19/07/2013		○	EPI566628	EPI566630	EPI566627	EPI566623	EPI566629	EPI566626	EPI566624	EPI566625	DRR051414
A/Myanmar/13M082/2013	Myanmar	2013	19/07/2013		○	EPI564868	EPI564870	EPI564867	EPI564863	EPI564869	EPI564866	EPI564864	EPI564865	DRR051415
A/Myanmar/13M084/2013	Myanmar	2013	19/07/2013		○	EPI566636	EPI566638	EPI566635	EPI566631	EPI566637	EPI566634	EPI566632	EPI566633	DRR051416
A/Myanmar/13M085/2013	Myanmar	2013	22/07/2013		○	EPI566644	EPI566646	EPI566643	EPI566639	EPI566645	EPI566642	EPI566640	EPI566641	DRR051417
A/Myanmar/13M087/2013	Myanmar	2013	22/07/2013		○	EPI566678	EPI585079	EPI566677	EPI566673	EPI566679	EPI566676	EPI566674	EPI566675	DRR051418
A/Myanmar/13M088/2013	Myanmar	2013	22/07/2013		○	EPI566686	EPI566688	EPI566685	EPI566681	EPI566687	EPI566684	EPI566682	EPI566683	DRR051419
A/Myanmar/13M089/2013	Myanmar	2013	22/07/2013		○	EPI566712	EPI566714	EPI566711	EPI566707	EPI566713	EPI566710	EPI566708	EPI566709	DRR051420
A/Myanmar/13M098/2013	Myanmar	2013	24/07/2013		○	EPI566720	EPI566722	EPI566719	EPI566715	EPI566721	EPI566718	EPI566716	EPI566717	DRR051421
A/Myanmar/13M102/2013	Myanmar	2013	24/07/2013		○	EPI566728	EPI566730	EPI566727	EPI566723	EPI566729	EPI566726	EPI566724	EPI566725	DRR051422
A/Myanmar/13M105/2013	Myanmar	2013	25/07/2013		○	EPI566736	EPI566738	EPI566735	EPI566731	EPI566737	EPI566734	EPI566732	EPI566733	DRR051423
A/Myanmar/13M109/2013	Myanmar	2013	25/07/2013		○	EPI566744	EPI566746	EPI566743	EPI566739	EPI566745	EPI566742	EPI566740	EPI566741	DRR051424
A/Myanmar/13M118/2013	Myanmar	2013	26/07/2013		○	EPI566752	EPI566754	EPI566751	EPI566747	EPI566753	EPI566750	EPI566748	EPI566749	DRR051425
A/Myanmar/13M124/2013	Myanmar	2013	16/07/2013		○	EPI566760	EPI566762	EPI566759	EPI566755	EPI566761	EPI566758	EPI566756	EPI566757	DRR051426
A/Myanmar/13M300/2013	Myanmar	2013	22/08/2013		○	EPI566768	EPI566770	EPI566767	EPI566763	EPI566769	EPI566766	EPI566764	EPI566765	DRR051427
A/Myanmar/14M039/2014	Myanmar	2014	24/06/2014		○	EPI568055	EPI568057	EPI568054	EPI568050	EPI568056	EPI568053	EPI568051	EPI568052	DRR051428
A/Myanmar/14M059/2014	Myanmar	2014	14/07/2014		○	EPI568063	EPI568065	EPI568062	EPI568058	EPI568064	EPI568061	EPI568059	EPI568060	DRR051429
A/Myanmar/14M122/2014	Myanmar	2014	08/07/2014		○	EPI568071	EPI568073	EPI568070	EPI568066	EPI568072	EPI568069	EPI568067	EPI568068	DRR051430
A/Myanmar/14M187/2014	Myanmar	2014	02/07/2014		○	EPI568079	EPI568081	EPI568078	EPI568074	EPI568080	EPI568077	EPI568075	EPI568076	DRR051431
A/Myanmar/14M240/2014	Myanmar	2014	04/08/2014		○	EPI568087	EPI568089	EPI568086	EPI568082	EPI568088	EPI568085	EPI568083	EPI568084	DRR051432
A/Myanmar/14M391/2014	Myanmar	2014	22/08/2014		○	EPI568095	EPI568097	EPI568094	EPI568090	EPI568096	EPI568093	EPI568091	EPI568092	DRR051433
A/Myanmar/14M465/2014	Myanmar	2014	08/09/2014		○	EPI568103	EPI568105	EPI568102	EPI568098	EPI568104	EPI568101	EPI568099	EPI568100	DRR051434
A/Vietnam/13V H3-2/2012	Vietnam	2012	28/12/2012		○	EPI567886	EPI567888	EPI567885	EPI567881	EPI567887	EPI567884	EPI567882	EPI567883	DRR051474
A/Vietnam/13V H3-3/2012	Vietnam	2012	23/07/2012		○	EPI567894	EPI567896	EPI567893	EPI567889	EPI567895	EPI567892	EPI567890	EPI567891	DRR051475
A/Vietnam/13V H3-9/2012	Vietnam	2012	27/07/2012		○	EPI567940	EPI567942	EPI567939	EPI567935	EPI567941	EPI567938	EPI567936	EPI567937	DRR051478
A/Vietnam/13V H3-10/2012	Vietnam	2012	25/07/2012		○	EPI567948	EPI567950	EPI567947	EPI567943	EPI567949	EPI567946	EPI567944	EPI567945	DRR051473
A/Vietnam/13V H3-4/2013	Vietnam	2013	13/04/2013		○	EPI567915	EPI567917	EPI567914	EPI567910	EPI567916	EPI567913	EPI567911	EPI567912	DRR051476
A/Vietnam/13V H3-8/2013	Vietnam	2013	25/09/2013		○	EPI567932	EPI567934	EPI567931	EPI567926	EPI567933	EPI567930	EPI567928	EPI567929	DRR051477
A/Lebanon/14L41/2014	Lebanon	2013-2014	09/01/2014		○	EPI580486	EPI580488	EPI580485	EPI580481	EPI580487	EPI580484	EPI580482	EPI580483	DRR051396
A/Lebanon/14L45/2014	Lebanon	2013-2014	22/01/2014		○	EPI580494	EPI580496	EPI580493	EPI580489	EPI580495	EPI580492	EPI580490	EPI580491	DRR051397
A/Lebanon/14L56/2014	Lebanon	2013-2014	31/01/2014		○	EPI580502	EPI580504	EPI580501	EPI580497	EPI580503	EPI580500	EPI580498	EPI580499	DRR051398
A/Lebanon/14L78/2014	Lebanon	2013-2014	10/02/2014		○	EPI580510	EPI580512	EPI580509	EPI580505	EPI580511	EPI580508	EPI580506	EPI580507	DRR051399
